# Reconfigurable Metasurface Antenna Based on the Liquid Metal for Flexible Scattering Fields Manipulation

**DOI:** 10.3390/mi12030243

**Published:** 2021-02-28

**Authors:** Ting Qian

**Affiliations:** Shanghai Technical Institute of Electronics and Information, Shanghai 200240, China; qianting_stiei@163.com

**Keywords:** liquid-metal metasurface, reconfigurable metasurface, reconfigurable antenna, beam manipulation

## Abstract

In this paper, we propose a reconfigurable metasurface antenna for flexible scattering field manipulation using liquid metal. Since the Eutectic gallium indium (EGaIn) liquid metal has a melting temperature around the general room temperature (about 30 °C), the structure based on the liquid metal can be easily reconstructed under the temperature control. We have designed an element cavity structure to contain liquid metal for its flexible shape-reconstruction. By melting and rotating the element structure, the shape of liquid metal can be altered, resulting in the distinct reflective phase responses. By arranging different metal structure distribution, we show that the scattering fields generated by the surface have diverse versions including single-beam, dual-beam, and so on. The experimental results have good consistency with the simulation design, which demonstrated our works. The presented reconfigurable scheme may promote more interest in various antenna designs on 5G and intelligent applications.

## 1. Introduction

The concept of metamaterials has attracted much attention in the past decade. Metamaterials are three-dimensional artificial structures with special electromagnetic properties. Due to the fact that metamaterials can be designed artificially, they can be widely used in a variety of applications, such as negative and zero refraction [1], perfect absorption [2,3,4], invisibility cloaking [5,6,7,8], dielectrics lenses [9,10] and vortex beams [11,12]. Reconfigurable metamaterials are composed of passive metamaterials and active components [13]. Its rapid development makes it possible to manufacture metadevices with practical functions and unique subwavelength devices [14,15,16,17,18,19]. At present, the tuning methods of reconfigurable metamaterials mainly include mechanical tuning [20,21], electronic tuning [22,23,24,25,26], material property tuning [27,28,29] and optical tuning [30,31].

The antenna is an essential part of the wireless information system. With the rapid development of wireless information technology, higher and higher requirements are put forward for the antenna system. It is expected that the antenna unit has the characteristics of a wide beam, wide frequency band and high gain. Therefore, people continue to explore new concepts and technologies of various antennas to break through the related problems. In recent years, the concept of reconfigurable antenna has been proposed. The reconfiguration of antenna performance is realized by adjusting the state of controllable devices integrated in the general radiation aperture. There are three methods to realize reconfiguration of antenna: electronic device reconfiguration (such as pin diode [32,33], radio frequency (RF) switches [34,35,36], varactor diode [37,38,39], etc.), mechanical reconfiguration [40,41] and changing the material properties of the antenna [42]. Through the reconfigurable technologies, the performance of antenna can be transformed, such as the working frequency [43,44,45], polarization mode [46,47,48], radiation pattern [49] and the combination of the above performance.

However, many of the researches we mentioned above need complex hardware system design, requiring active components like PIN diodes, switches, and varactors, demanding high control complexity and cost. To explore a flexible and low-cost reconfigurable method, here, we present a metasurface antenna based on liquid metal for flexible scattering fields manipulation. A square-ring cavity structure has been designed, simulated and fabricated to demonstrate our design. By rotating and reconstructing the liquid metal shape, the reflected phase response of the element shows various distinct states, covering more than 270° phase range. We designed several phase distributions on the surface to generate the scattering beam in different directions. The measured results have good agreement with the simulation, verifying our design. We believe this work will stimulate more researches on reconfigurable and flexible designs utilizing liquid metal for diverse applications.

## 2. Principle and Design

**Figure 1:**
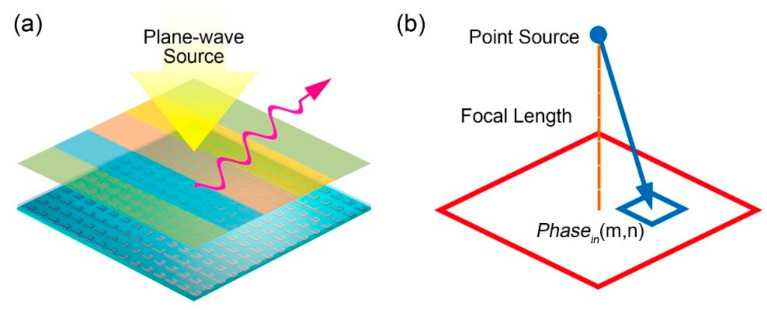
The schematic of the presented reconfigurable metasurface antenna. (**a**) The anomalous refraction under plane-wave incidence. (**b**) The incident phase calculation of the point source feed.

As shown in Figure 1, we presented a metasurface antenna system composed of a reflected surface with reconfigurable liquid metal on it and a feed antenna. When the incident wave from the feed antenna is reflected by the specific metal structure, the desired phase distribution is generated, as illustrated in Figure 1. According to the length from the phase center of the antenna to the different elements, the relevant phase distribution on the surface is obtained easily according to the equation below, as we exhibited in Figure 1b.

(1) $$Phase_{in}\left(m,\;n\right)=\frac{2\pi L\left(m,\;n\right)}{\lambda }$$

where L(m, n) means the length from the phase center of feed antenna to the element as *m*th row and *n*th column, λ is the wavelength of the operating frequency. To design the desired scattering pattern, we first calculate the phase distribution $Phase_{de}$ of the specific beam scattering fields, such as beam-deflection or dual-beam fields. Then the final phase distribution can be calculated following Equation (2) as below.

(2) $$Phase_{final}=Phase_{de}-Phase_{in}$$

To simplify the phase states, we designed the 2-bit reflective states as 0°, 90°, 180°, and 270° to establish the final surface phase response. The final phase distribution is generated by liquid-metal elements with different shapes, which can be achieved by heating and cooling at a certain temperature. By arranging the various phase distributions produced by the liquid-metal elements, we can realize various radiation patterns like single-beam, dual-beam, and three-beam.

**Figure 2:**
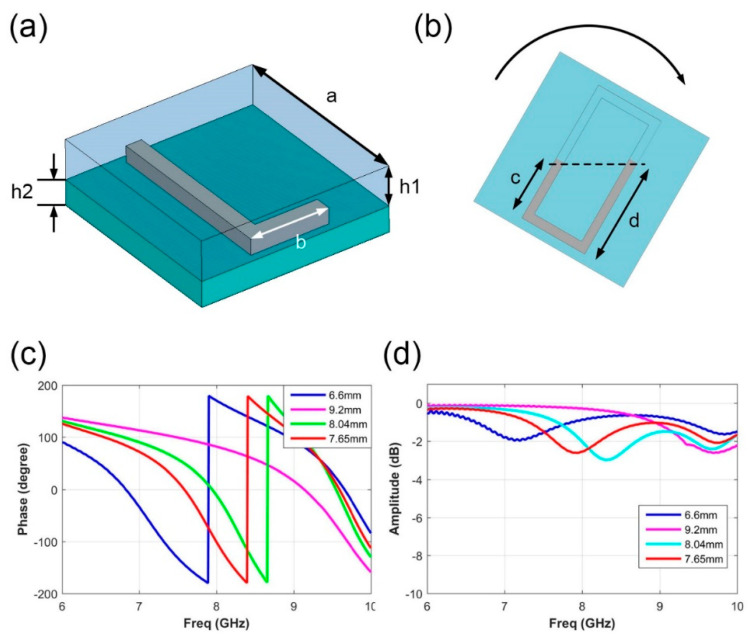
The element design and its simulation performance. (**a**) The detailed structure of the metasurface element. (**b**) The liquid-metal reconstruction by rotating the element. (**c**) The simulated results of reflection phase responses of four element dimensions, where d is 6.6 mm, 9.2mm, 8.04 mm, 7.65 mm. (**d**) The simulated results of reflection amplitude responses of four element dimensions, where d is 6.6 mm, 9.2 mm, 8.04 mm, and 7.65 mm.

To achieve the flexible phase-reconfigurable capability, we designed a metasurface element with a rectangular-ring cavity to contain the liquid metal and guide it into the desired shape. The detailed element structure is presented in Figure 2a. In the upper layer, the cavity structure of the element is polyamides within three-dimensional printing technology, whose permittivity is 3. Eutectic gallium indium (EGaIn) alloy with a melting point around room-temperature is employed for the desired shape reconstruction. The designed dimension of the element structure is provided as follows: a = 12 mm, b = 5mm, c+d = 12 mm, h1 = 2.5 mm, h2 = 1.6 mm. The alloy shape reconstruction method was first melted under a heating temperature a little higher than its melting point. Then we rotated the element at a certain angle to make the liquid metal flow into the specific size, as shown in Figure 2b. After the alloy reaches the target dimension, we cool the metal to fix its shape. To simplify the control states of liquid metal, we design four states with the liquid metal length as follows: (1) d = 6.6 mm, (2) d = 9.2 mm, (3) d = 8.04 mm, (4) d = 7.65 mm. These four states relate to the 2-bit phase responses as 0°, 90°, 180°, and 270°, theoretically. To simply express the phase states of these four responses, we encode them as 0, 1, 2, and 3, respectively. Figure 2c,d list the simulated phase and amplitude response for reflection wave. The element simulations are performed in a commercial software, CST microwave studio. The periodic boundary condition and linear polarization incidence are applied in the simulations. From the curve data, we can clearly observe that the reflected amplitude responses of four states are all above −3 dB, promising a fair reflection efficiency under plane wave incidence. It should be noted that the reflection efficiency can be further improved by applying the material with lower loss. The phase responses are provided in Figure 2c, in which the four states are labeled in different colors, respectively. The simulated four states exhibit 170.2°, 81.8°, −11.4°, −101.1° at 8 GHz, whose phase difference is about 90°. To indicate the phase arrangement on the reflecting surface clearly, we have used the phase code “0”, “1”, “2”, and “3” to represent these four phase states.

**Figure 3:**
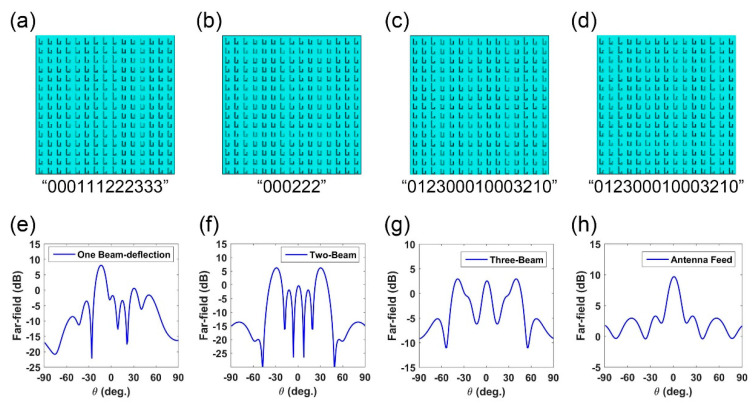
The designed metasurface schemes for different beam manipulation and the related simulation results for far-fields. (**a**–**d**) The metasurface elements configuration for different surface phase distributions, in which the phase code along *x*-axis is “0001112223333”, “012300010003210”, “000222” and “012300010003210”, respectively. Please note that the phase distributions are the same in (**b**,**d**), but their excitations are plane-wave and antenna-source, respectively. The simulated far-field results for the related phase schemes are listed in (**e**–**h**), where (**e**–**g**) are excited by plane-wave and (**h**) is excited by antenna.

The architecture of the presented metasurface antenna is already shown in Figure 1. A metasurface array composed of 15 × 15 elements is designed and simulated in CST. Two feed source schemes are applied to demonstrate the scattering field manipulation performance. One is plane-wave excitation while the other applies micro-strip antenna as feed, as showed in Figure 1a,b, where the focal length of the antenna feed is set as 60 mm. For the plane wave incidence, we design three phase distributions to realize beam-deflection, dual-beam, and trinal-beam, respectively, as listed in Figure 3a–c. For the point source incidence, we designed a phase distribution, as shown in Figure 3d, to obtain a nearly consistent phase distribution on the reflection surface. To demonstrate their performance, we performed the full-wave simulation in CST to test the scattering fields. The related far-fields simulation data is presented in Figure 3e–h, in which the open boundary condition is applied in all schemes. In Figure 3a–c, four phase distributions are designed as “0001112223333”, “000222”, and “012300010003210”. In Figure 3a, the single beam scattering direction is at about 20° based on the gradient phase distribution. For the dual-beam scattering field, the two main beams point at about 30° when applying phase code “000222”. As the designed metasurface has a 15 × 15 unit array, the code “000222” fills the array as “00022200022200” along the *x*-axis while keeping the same phase state along the other direction (*y*-axis). Other phase codes follow the same arranging method. The phase distributions in Figure 3c,d are the same, but their excitation feeds are different. Figure 3g presents the far-field result under plane-wave illumination, where a three-beam scattering field is clearly observed. Figure 3h presents the far-field result under micro-strip antenna feeding, in which the beam gain is about 10 dB.

**Figure 4:**
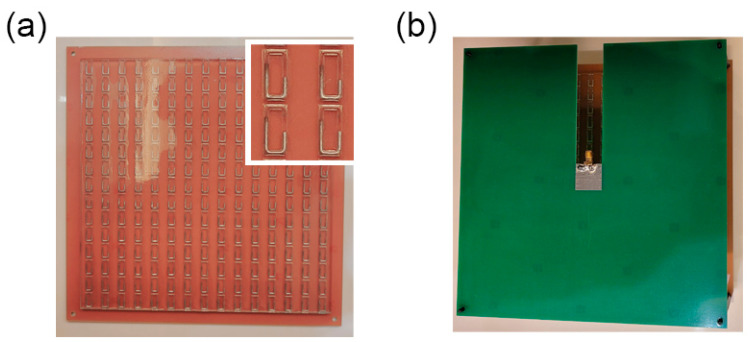
The fabricated metasurface antenna sample photograph. (**a**) Photograph of the liquid-metal metasurface sample as well as its enlarged figure of the element. (**b**) The assembled metasurface antenna fed by a micro-strip antenna.

In the experimental verification, we fabricated a 15 × 15 metasurface sample to demonstrate the design we presented above. Since the metasurface is composed of two substrate layers, we fabricated the bottom substrate layer and upper polyamides layer into one piece. The bottom substrate, FR4, and the bottom metal layer are all fabricated together into one board within Printed Circuit Board (PCB) technology. The transparent polyamides containing liquid metal is fabricated based on 3D printing technology, where the rectangular-ring cavity is sculptured on the substrate. Each cavity is injected with a certain amount of melted EGaIn alloy to construct the designed metal structure. After the liquid metal solidifies, we tightly glue the two dielectric plates together. It is worth mentioning that single element manipulation is possible if we can use a point heater to melt one element while keeping the surrounding elements at a low temperature. The most time-consuming part is metal shape reconstruction, which mainly depends on the melting time of the liquid metal. In our experiment, the melting time of liquid metal is about 30 s. For a large array modulation, we can apply a fast heating and cooling device to arrange the phase-pattern distribution more quickly. Some other solutions such as phase-pattern optimization reduce the amount of the reconfigurable element each time. By applying several fast heating and cooling devices, we estimate that the melting time can be reduced to less than 10 s, and the average time of the metasurface pattern reconfiguration can be limited to about three minutes. The fabricated sample picture is provided in Figure 4a, in which an enlarged element figure is given. For plane-wave excitation schemes, the metasurface is directly illuminated by a high-gain horn at long distance, to imitate an approximate plane-wave incidence. For microstrip antenna excitation, we fabricated another PCB plate to excite the metasurface. The antenna was set at the central axis and about 60 mm away from the surface. The antenna PCB plate was fixed with the liquid-metal metasurface using polyamide screws, as showed in Figure 4b. The far-field measurement was performed in a standard microwave chamber room. A two-dimensional far-field of the metasurface was tested based on a plane turning table.

**Figure 5:**
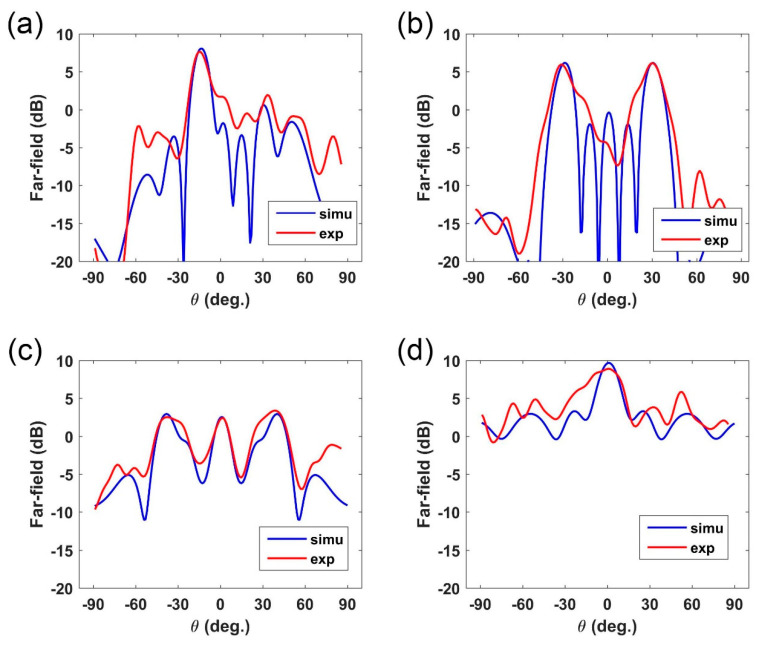
The measured far-field results for the designed four-phase distributions, as well as the related simulation data for comparison. (**a**–**c**) The measured far-field results for plane-wave incidence to realize one-beam, dual-beam, and three-beam. (**d**) The single beam reflection under antenna excitation.

The measurement results are given in Figure 5, in which we perform three phase distributions to respectively demonstrate single-beam, dual-beam, three-beam under plane-wave, and point source excitation. From the measured data, we can clearly observe the single-beam deflection, dual-beam, and three-beam, respectively, in Figure 5a–c when under plane-wave illumination. For microstrip antenna feed, the tested result is showed in Figure 5a, where the gain of the main beam is about 10 dB. Compared to the previous works [15,16,17], the directivity of the presented four schemes in this work is about 10% lower, resulted from the substrate loss of FR4. But this could be improved by using the high-quality substrate with lower loss. Comparing the measured results and simulated results, we can observe a slight difference in the scattering angle of the main beams. The scattering energy in other directions also shows the discrepancy between simulations and experiments. The reasons for these differences mainly include: (1) the fabrication error in polyamides cavity support and manual assembling, which may lead to the error in liquid-metal dimensions; (2) The non-ideal plane wave source from the horn antenna; (3) The manual operation in experimental measurements; (4) The phase center error between simulation and measurements.

## 3. Conclusions

In summary, we present a reconfigurable metasurface antenna using EGaIn alloy to realize flexible scattering fields manipulation. By changing the metal shape of the metasurface element along the polarization direction, the reflected element phase responses show distinct phase differences. We designed a rectangular-ring cavity to contain and guide the liquid metal into the desired shape. An array of 2-bit phase responses (0°, 90°, 180°, and 270°) were achieved using four dimensions of alloy length. Since the EGaIn alloy has a melting point of around 30 ℃, the alloy is easily melted under slight heating and reconstruct into the designed size. Comparing to the idea proposed in Lei Chen’s work [50], this work presents a more powerful phase-manipulation method that can achieve not only 2-bit phase states but also almost continuous phase-manipulation in one single element. The rectangular ring structure presented in this paper exhibits a more flexible phase-manipulation capability with a large modulation range. All elements were fabricated into one metasurface configuration, apparently improving its integration level. Four phase distributions are designed for plane-wave and microstrip antenna excitation to obtain single-beam deflection, dual-beam, and three-beam, respectively. We performed the experimental verification for three-phase distribution designs and obtained good agreement with the simulation data. Comparing to the active reconfigurable methods such as utilizing PIN diodes and varactors, the liquid-metal metasurface antenna presented in this work has much less system complexity (no other control unit), lower cost (generally one-tenth of other methods), and lower power consumption (no operating current). The flexible and powerful manipulation control of liquid metal shows good performance and potential in the next generation of wireless communication applications and smart scenarios for microwave scattering manipulation.
